# Supplementary material for: Complex‐centric proteome profiling by SEC‐SWATH‐MS
Source: Mol Syst Biol. 2019 Jan 14;15(1):e8438. doi: 10.15252/msb.20188438 (PMC6346213; doi:10.15252/msb.20188438)
Supplement: Supplementary file 7 — Dataset EV6 [file MSB-15-e8438-s007.zip › feature_plots_bioplex/Q13595.pdf]

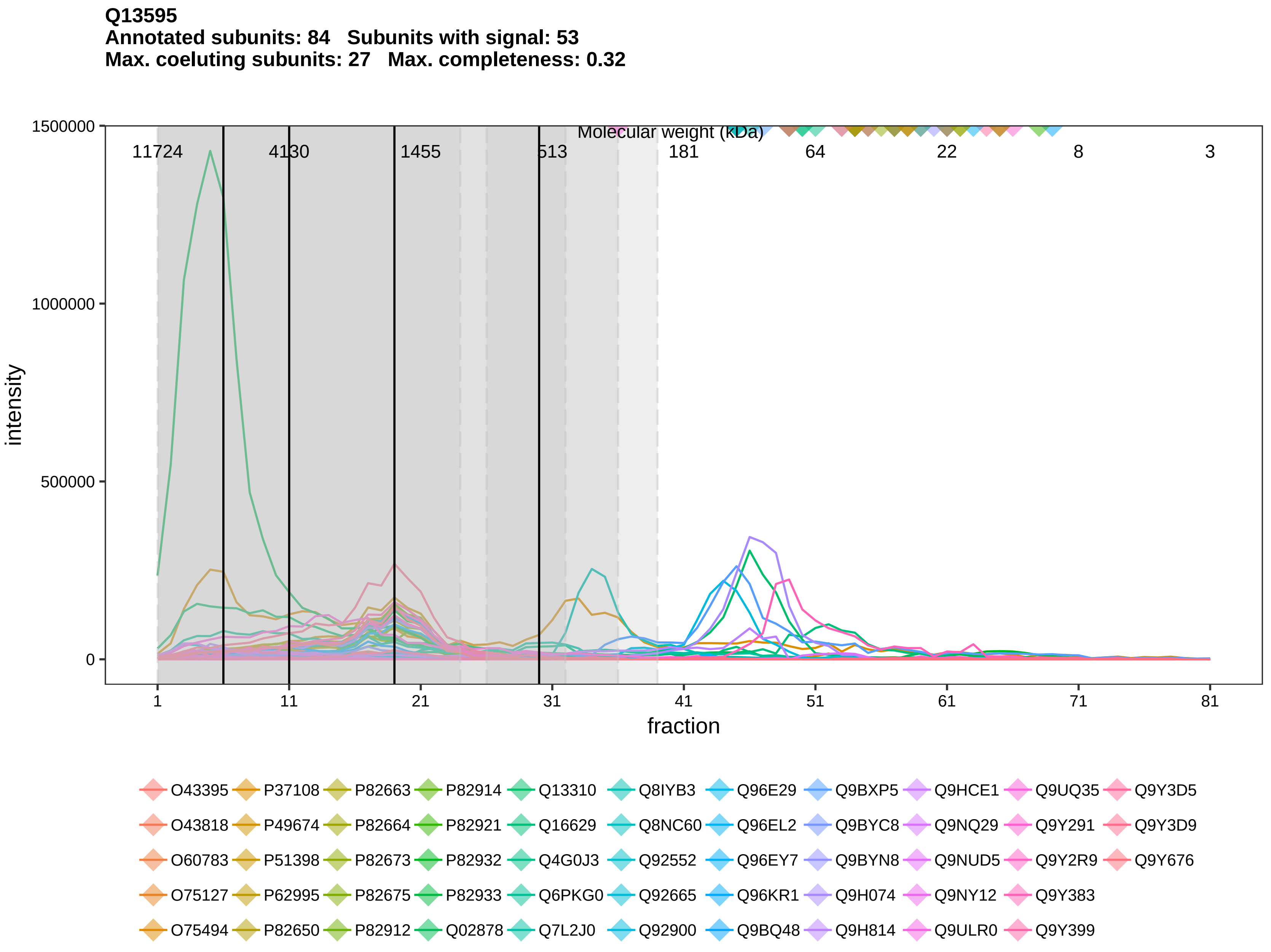
Q13595
Annotated subunits: 84 Subunits with signal: 53
Max. coeluting subunits: 27 Max. completeness: 0.32
Molecular weight (kDa)
11724
4130
1455
513
181
64
22
8
3
intensity
1500000
1000000
500000
0
fraction
1
11
21
31
41
51
61
71
81
O43395
O43818
O60783
O75127
O75494
P37108
P49674
P51398
P62995
P82650
P82663
P82664
P82673
P82675
P82912
P82914
P82921
P82932
P82933
Q02878
Q13310
Q16629
Q4G0J3
Q6PKG0
Q7L2J0
Q8IYB3
Q8NC60
Q92552
Q92665
Q92900
Q96E29
Q96EL2
Q96EY7
Q96KR1
Q9BQ48
Q9BXP5
Q9BYC8
Q9BYN8
Q9H074
Q9H814
Q9HCE1
Q9NQ29
Q9NUD5
Q9NY12
Q9ULR0
Q9UQ35
Q9Y291
Q9Y2R9
Q9Y383
Q9Y399
Q9Y3D5
Q9Y3D9
Q9Y676
